# Hydrologic Conditions Describe West Nile Virus Risk in Colorado

**DOI:** 10.3390/ijerph7020494

**Published:** 2010-02-11

**Authors:** Jeffrey Shaman, Jonathan F. Day, Nicholas Komar

**Affiliations:** 1 College of Oceanic and Atmospheric Sciences, Oregon State University, 104 COAS Administration Building, Corvallis, OR 97330, USA; 2 Florida Medical Entomology Laboratory, Institute of Food and Agricultural Sciences, University of Florida, 200 9^th^ Street SE, Vero Beach, FL 32962, USA; E-Mail: jfda@ufl.edu; 3 Arbovirus Diseases Branch, Centers for Disease Control and Prevention, 3150 Rampart Road, Fort Collins, CO 80521, USA; E-Mail: nck6@cdc.gov

**Keywords:** West Nile Virus, hydrology, transmission, amplification, spatial autocorrelation, *Culex* mosquitoes

## Abstract

We examine the relationship between hydrologic variability and the incidence of human disease associated with West Nile virus (WNV; family *Flaviviridae*, genus *Flavivirus*) infection (hereafter termed “human WN cases”) in Colorado from 2002 to 2007. We find that local hydrologic conditions, as simulated by the Mosaic hydrology model, are associated with differences in human WN cases. In Colorado’s eastern plains, wetter spring conditions and drier summer conditions predict human WN cases. In Colorado’s western mountains, drier spring and summer conditions weakly predict human WN cases. These findings support two working hypotheses: (1) wet spring conditions increase the abundance of *Culex tarsalis* vectors in the plains, and (2) dry summer conditions, and respondent irrigational practices during such droughts, favor *Cx. pipiens* and *Cx. tarsalis* abundance throughout Colorado. Both of these processes potentially increase the local vector-to-host ratio, favoring WNV amplification among competent avian hosts and bridging to humans.

## 1. Introduction

Hydrologic conditions, including precipitation and land surface wetness, are known to affect mosquito-borne disease transmission [1–5]. The first three stages of the mosquito life cycle (egg, larvae, and pupae) are aquatic. Consequently, mosquito abundance and the transmission of mosquito-borne pathogens can be affected by hydrologic variability, in particular fluctuations in the water cycle that alter the quality and availability of mosquito breeding habitats.

Precipitation is the principal source of water at the land surface. Precipitation in the form of rain and snow directly moistens the land surface, increases river flow, and expands riparian zones. The degree to which these hydrologic conditions are linked across the land surface, *i.e.*, spatially autocorrelated, is greatly constrained by the spatial scale of precipitation-producing storm systems.

The spatial scales of storms are highly dependent on latitude. Tropical storms typically develop at geographic length scales ranging from ≤1–200 km. Such systems include small-scale convective cells and larger mesoscale structures, including cyclones. These smaller storm scales generate strong spatial variations in local measured rainfall and soil moisture.

At middle and high latitudes, storm systems typically develop at length scales greater than 200 km. These larger storms are referred to as synoptic weather systems and can produce precipitation over very large areas (e.g., the entire eastern continental U.S.). As a consequence, hydrologic conditions, including precipitation and soil moisture, can be highly spatially autocorrelated at these larger, synoptic length scales. Smaller length-scale variability of these conditions also will develop due to variations in synoptic storm structure, intensity, and translational speed. In addition, smaller length scale storms, such as summer convective thunderstorms, can produce smaller-scale heterogeneity in hydrologic conditions at middle and high latitudes.

Human WN cases were first reported in Colorado during 2002. In 2003, a major outbreak of WNV took place in Colorado with 2,947 human cases reported. Since 2003, annual human WN cases in Colorado have numbered in the hundreds (Table 1). Hydrologic conditions influence vector biology, ecology and vector-borne disease incidence and may be useful for predicting the severity of impending WNV outbreaks. Accordingly, we examine the relationship between local hydrologic variability, as simulated at 0.125° resolution by a land surface hydrology model, and the incidence of reported human WNV infections in Colorado. From this statistical analysis we infer the effects of hydrologic conditions in Colorado on mosquito population dynamics, WNV amplification, and transmission of WNV to humans.

## 2. Materials and Methods

### 2.1. Mosaic Hydrology Model

We employed simulations of hydrologic conditions using the Mosaic hydrology model [6]. This hydrology model divides the land surface into square grid cells, each with a multilayer soil column structure. In response to local meteorological forcing, the Mosaic model computes column-averaged water and energy fluxes from the land surface. Each soil column cell is further divided into a ‘mosaic’ of tiles, each representing different vegetative surfaces, which account for variability of surface characteristics within each cell. Observed vegetation distributions are used to determine the partitioning of tiles, and the water and energy balances in each tile are simulated independently.

Mosaic model simulations are produced through the North American Land Data Assimilation Systems (NLDAS) project, and are available in hourly time steps at 0.125° resolution from October 1996 through the present [7,8]. At this resolution (approximately 13 km by 13 km), each grid cell resolves hydrologic conditions at a geographic scale matching the upper limit of the flight range reported for *Culex tarsalis* in California [9] and represents an area in which the vector mosquito population and WNV transmission dynamics are localized. The Mosaic model uses three soil layers with thicknesses from top to bottom of 10, 30, and 160 cm, respectively, and a uniform rooting depth of 40 cm. Water storage in each model column layer is the weighted average of the water storage from the column tiles.

For this work, we used Mosaic model simulations of root zone soil moisture (RZSM) as our measure of land surface wetness in Colorado. These estimates represent water content in the top 40 cm of the soil column and are made in units of kg/m^2^. The hourly RZSM estimates were aggregated to monthly averages during the period 2001–2007 and compared with the spatio-temporally matched occurrence of per capita human WN cases.

**Figure 1.**
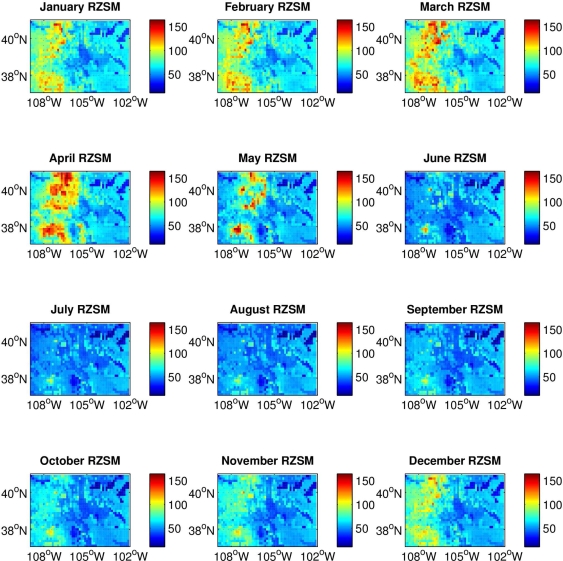
Climatology of monthly Mosaic model root zone soil moisture (RZSM) simulated conditions (2001–2007) throughout Colorado. RZSM estimates are units of kg/m^2^. The mountainous western portion of Colorado has a larger seasonal cycle with greater variations in RZSM due to the large amounts of snowfall that typically occur there in late fall, winter and early spring.

RZSM content varies across Colorado, with the western mountainous portion of the state much wetter during late fall, winter and early spring and possessing a larger amplitude annual cycle than the eastern plains (Figure 1). This climatological east-west difference in RZSM conditions is due to greater snowfall and the development of a snowpack over the Rocky Mountains in western Colorado during colder months. Because of this difference, we conducted separate analyses for the high plains of eastern Colorado and the mountains of western Colorado. This partitioning is further justified by differences between these two regions in other environmental conditions, such as temperature and vegetation, which also can affect vector mosquito and avian host distribution, abundance and activity. Eastern Colorado was defined as grid locations from 105.5 °W to 102 °W; western Colorado was defined as grid locations from 109 °W to 106 °W. To more clearly delineate the hydrologic differences between eastern and western Colorado a half-degree gap at the border between the two domains (between 106 °W and 105.5 °W) was omitted from the analysis.

### 2.2. Human WN Case Data

Human WN case data for 2002–2007 were provided by the Colorado Department of Public Health and Environment, Communicable Disease Epidemiology Program. These data are organized by onset date of illness and census tract of the individual and include cases of WN fever and WN neurological disease. Census tracts are subdivisions of a county averaging about 4,000 people. Some counties are themselves a single census tract, whereas within municipalities, census tracts represent much smaller areas.

Our use of the census tract data includes assumptions that infections were acquired within the census tract of residence of the individual and that reporting rates were consistent. The latter assumption was confirmed by examination of the ratio of WN fever to WN neurological disease cases; these were found to be consistent among Colorado counties through time.

The human WN case data were temporally aggregated to provide monthly totals of human WN cases for each county. Census tract population data were derived from U.S. Census Bureau sources and used to determine monthly case rates within each census tract on a per capita basis. The location of each census tract was specified geographically by its centroid. A number of human WN cases (n = 75, <2%) were only identified to county. In these instances, these extra cases were specified geographically by their county centroid and the monthly per capita rates at the county centroids were calculated using county population data.

**Figure 2.**
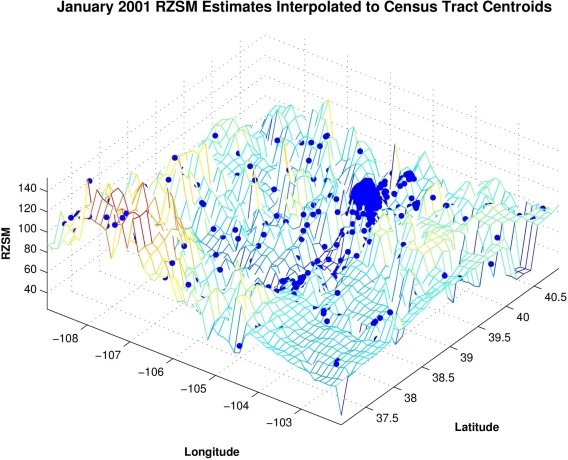
Plot of the 0.125 resolution RZSM estimates for January 2001 and the interpolation of this gridded surface to the census tract and county centroids (blue dots).

To facilitate our analyses, the monthly 0.125° grid resolution RZSM estimates were interpolated to all census tract and county centroids using a triangle-based cubic fitting (Figure 2). This interpolation provides RZSM estimates at each of the 1,126 centroids in Colorado and permits direct comparison of hydrology and human WN case rates. The analyses (described below) were also repeated using human WN case data interpolated to the 0.125° grid resolution of the RZSM estimates. The results using this latter interpolation to the RZSM grid matched those of the interpolation to the census tract and county centroids and are not shown.

### 2.3. Study Areas

The eastern Colorado high plains are predominantly grasslands interlaced by river riparian zones dominated by willow and cottonwood trees (family *Salicaceae*) [10]. These riparian zones provide habitat for both mosquito vectors and avian hosts, and have been the loci of WNV activity in eastern Colorado. The primary vectors of WNV in eastern Colorado are *Cx. tarsalis* and *Cx. pipiens* [11]. *Culex tarsalis* prefers clean water (*i.e.*, less eutrophic) larval habitats [12], is an opportunistic blood feeder, and has high vector competence [13,14], whereas *Cx. pipiens* prefers eutrophic larval habitats, is more aviaphilic [15], and is moderately vector competent [16]. These differences in the oviposition, blood feeding, and vector competencies of *Cx. tarsalis* and *Cx. pipiens* may produce different biological responses to environmental and hydrologic variability.

The habitat of western Colorado is dominated by the Rocky Mountain alpine landscape. A large snowpack develops in winter (Figure 1), which feeds rivers flowing both east and west throughout the year. Vegetation is more cold tolerant at higher elevations with large tracts of alpine tundra, subalpine forests of spruce (*Picea pungens, Picea engelmannii*), fir (*Abies lasiocarpa, Pseudotsuga menziesii*) and lodgepole pine (*Pinus contorta*) forest, and montane forests of ponderosa pine (*Pinus ponderosa*), quaking aspen (*Populus temuloides*) and Gambel oak (*Quercus gambelii*) [10]. As in eastern Colorado, willow and cottonwood trees line the riparian corridors, and the primary vectors of WNV are *Cx. tarsalis* and *Cx. pipiens.* Vector activity occurs primarily in the populated valleys at lower elevation, diminishing substantially in areas of higher elevation (e.g., >3,000 m) where colder temperatures prevail [17].

### 2.4. Grouping Analysis

To explore a potential association between the year-to-year variability of hydrological conditions and human WN case incidence, sites were categorized on a yearly basis by the per capita number of human WN cases at each grid point. To perform the categorization, a threshold was established (e.g., 100 or more human WN cases per 100,000 people at a site for a given year). Monthly RZSM estimates for all site-years meeting this criterion were placed in Group 1, and those site-years failing to meet this criterion were placed in Group 2. Once completed, both groupings possessed an empiric distribution of monthly hydrologic conditions associated with a category of human WN cases (e.g., 100 or more human WN cases per 100,000 people at a site for a given year for Group 1; fewer than 100 human WN cases per 100,000 people at a site for a given year for Group 2), which were compared graphically. This grouping analysis was used to guide the regression analysis; however, mean differences between Group 1 and 2 were assessed using Student’s t test.

### 2.5. Statistical Analysis

To formally assess the effect of hydrologic variability on human WN cases, we used simultaneous autoregression (SAR) with a generalized linear model (GLM) [18,19]. This continuous model is of the form:

(1) ÿ=αW(ÿ−Xβ)+Xβ+ε

where **ÿ** are the total yearly number of per capita human WN cases at each grid site, **X** are monthly RZSM estimates at those grid sites, **W** is a matrix of weights whereby each grid-year value is modeled not only as a function of RZSM conditions, but also as a weighted sum of the residuals of neighboring grid-year values, scaled as a function of distance, *α* and *β* are parameter estimates, and *ɛ* is the model error. The human WN case data possess an overdispersed Poisson distribution. Therefore, for the GLM, the Poisson distribution was used with a dispersion parameter to account for the inflated variance.

The SAR framework explicitly accounts for spatial dependence within the human case data for improved parameter estimation. The effectiveness of the SAR framework is assessed through examination of the spatial autocorrelation among model residuals using the Moran I statistic. Significance of the Moran I statistic is assessed by comparing this value to a distribution of Moran I statistics calculated for repeated random permutations of the model residuals in space.

The SAR GLM and Moran I statistics were computed for a number of weighting matrices (**W**), including inverse distance, inverse distance squared, and inverse distance cubed. The inverse distance matrix presented here provides the best model fit, as demonstrated by lower model deviance, stronger parameter estimates with reduced standard error, and Moran I statistics closer to the expected value, −1*/*(*n* − 1), where *n* is the number of site-years.

## 3. Results

**Figure 3.**
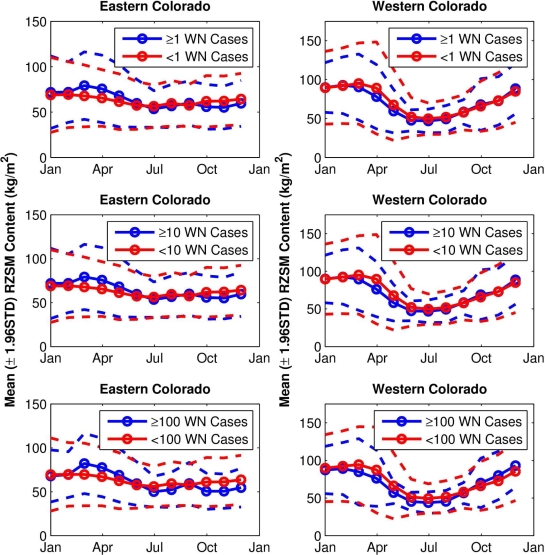
Mean annual monthly Mosaic model estimates of hydrology (RZSM in kg/m^2^) during 2002–2007 for: top left) eastern Colorado (105.5−102.0 °W) site-years with 1 or more human WN cases per 100,000 people (n = 977) and site-years with fewer than 1 human WN cases per 100,000 people; middle left) eastern Colorado site-years with 10 or more human WN cases per 100,000 people (n = 969) and site-years with fewer than 10 human WN cases per 100,000 people; bottom left) eastern Colorado site-years with 100 or more human WN cases per 100,000 people (n = 251) and site-years with fewer than 100 human WN cases per 100,000 people. The right column shows the same groupings for western Colorado (109.0−106.0 °W) with respective Group 1 numbers of n = 178, 168 and 26 from top to bottom. Corresponding 2 standard error intervals for each grouping are indicated by the color-coded dashed lines. For all plots the blue lines show the grouping of monthly RZSM associated with higher human WN case rates (Group 1).

The grouping analyses show differences in the seasonal evolution of hydrological conditions between sites with higher numbers of human WN cases and lower numbers of human WN cases. In eastern Colorado (Figure 3, left column), RZSM conditions are wetter from March to June for the Group 1 site-years (higher numbers of human WN cases, the blue lines) than for the Group 2 site-years (fewer human WN cases, the red lines, p < 0.0001 for all, except June with a categorization of 100, where p < 0.05). In addition, average RZSM conditions were drier for July–December for site-years with higher numbers of human WN cases (p < 0.0001 for all, except September with a categorization of 100, where p < 0.05). This seasonal change in hydrologic conditions favoring human WN cases is evident for all grouping categorizations (*i.e.*, 1, 10, 100, *etc*.), suggesting that a combination of wet spring and dry summer increases risk of human WNV infection in eastern Colorado.

**Figure 4.**
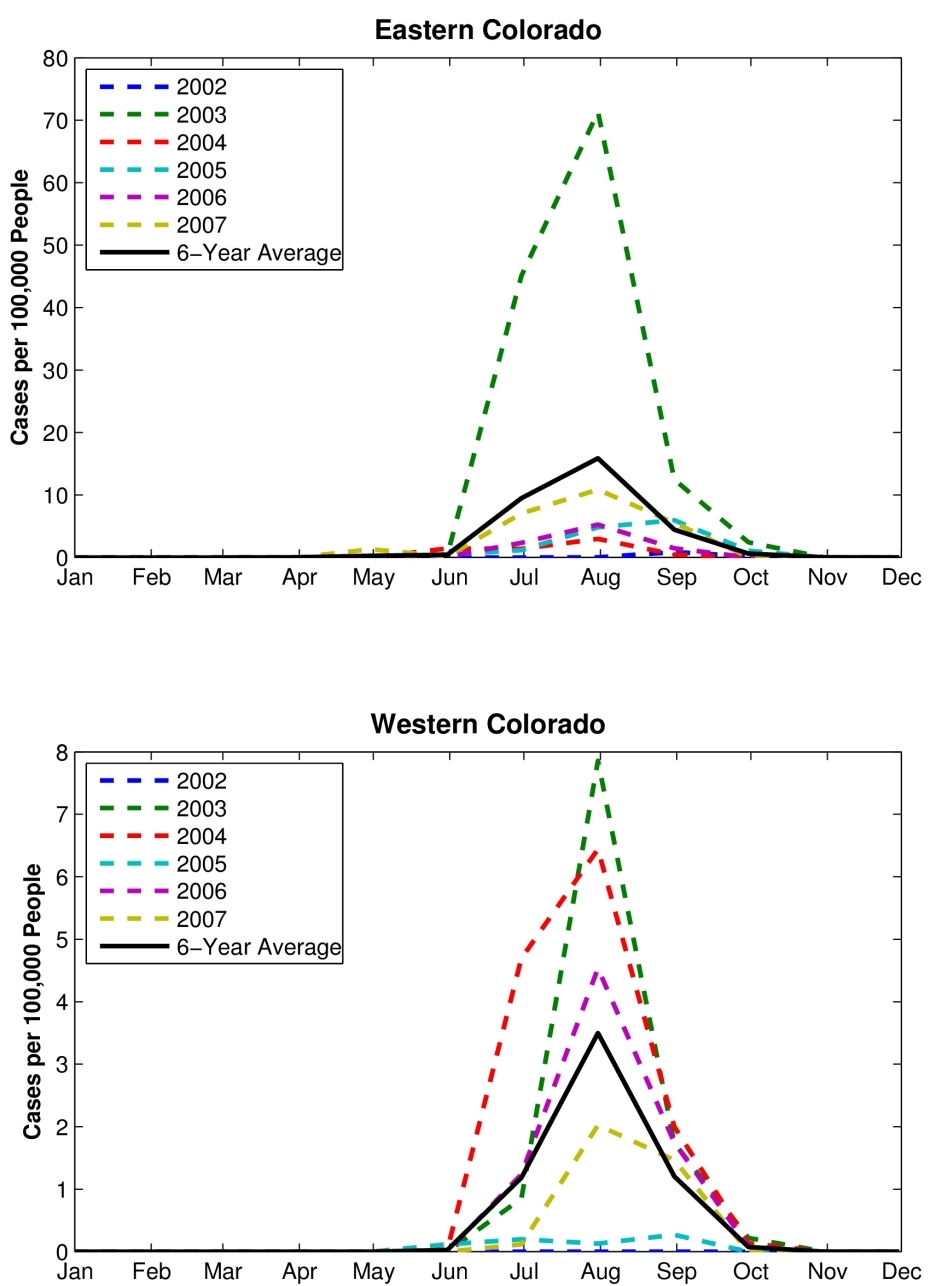
Monthly per capita human WN cases during 2002–2007 in eastern Colorado (105.5−102.0 °W) and western Colorado (109.0−106.0 °W).

In western Colorado (Figure 3, right column), RZSM conditions were drier during March–August for Group 1 site-years (p < 0.05). Western Colorado RZSM conditions were also wetter during October-December at site-years with higher numbers of human WN cases; however, this shift from dry to wet conditions takes place after the WNV transmission season, which peaks in August (Figure 4), and thus does not impact human WN cases. For western Colorado, dry spring and summer appears to increase risk of human WNV infection.

**Figure 5.**
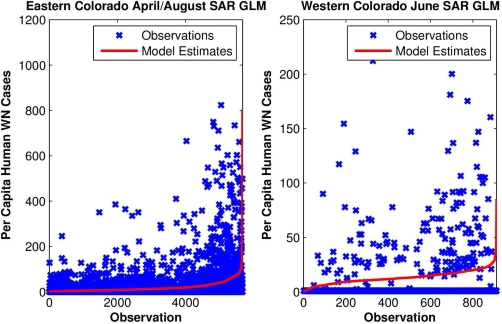
Gridded monthly per capita human WN cases and corresponding SAR GLM best-fit estimates of monthly per capita human WN cases during 2002–2007 in eastern and western Colorado modeled as a function of local monthly RZSM conditions within that year. The observations and model estimates are ordered in pairs by the latter variable, from least to greatest.

To test these hypotheses further, yearly per capita numbers of human WN cases at each site were regressed upon local monthly RZSM conditions. For eastern Colorado, both spring and summer RZSM conditions were included in the model (e.g., March and July, April and August, *etc*.; Figure 5). These models also indicated that local wet spring and dry summer conditions correlate positively with human WN cases (Table 2). For western Colorado, either spring or summer conditions were included in a univariate model and indicate that dry conditions are associated with human WN cases (Table 3).

## 4. Discussion

West Nile virus is the most widely distributed arbovirus on earth. However, the environmental conditions controlling rates of WNV amplification and transmission are poorly understood. Regional differences among habitat type, habitat fragmentation, human land use practices, climate, hydrology, vector species composition, and vertebrate reservoir host species composition exist throughout its distribution. As a consequence, the factors driving WNV amplification and transmission likely differ regionally.

In Colorado, local soil moisture conditions are associated with human WN cases. Specifically, in eastern Colorado wetter than usual spring and drier than usual summer conditions predict increased human WN cases. In western Colorado the results are less robust, but drier than usual spring and summer conditions may be associated with increased human WN cases. Hence, WNV dynamics are more closely tied to hydrologic variability in the more arid eastern Colorado ecosystem.

### 4.1. Localized Amplification and Transmission

For high levels of WNV transmission to humans to occur in a locality, vector mosquitoes must acquire the virus from an infectious vertebrate reservoir host (typically a passerine bird), survive the extrinsic incubation period of the virus, and transmit the virus to additional susceptible reservoirs, which, in turn, infect additional vectors. Through this detailed and highly localized amplification process, a portion of the infectious, host-seeking vectors will encounter incidental hosts, such as humans and horses, resulting in epidemic or epizootic transmission.

The localized nature of the WNV amplification process [20] implies that the high numbers of human WN cases reported in Colorado during 2003 resulted from many localized, mostly independent, amplification events. Vector populations involved in amplification would have been influenced by local hydrologic conditions. Hence, for this analysis it was important to resolve hydrologic conditions at the spatial scales at which amplification occurs. The RZSM grid cell estimates employed here represent areas 0.125° by 0.125° (approximately 13 km by 13 km). Thus, each grid cell resolves hydrologic conditions at a geographic scale matching the upper limit of the reported *Culex tarsalis* flight range in California [9] and represents an area in which the vector mosquito population and amplification dynamics are localized.

### 4.2. Biological Basis

The mechanisms that determine how land surface wetness affects WNV amplification and transmission in Colorado remain to be fully understood. Amplification of WNV is favored by an increase in the vector-to-host ratio [21,22]. Hydrologic conditions can facilitate such an increase in this ratio by simply increasing vector abundance, or by geographically concentrating vectors around limited water resources already exploited by avian hosts [4,5].

During wet springs in eastern Colorado, we speculate that the increased land surface wetness produces a greater abundance of mosquito breeding habitats. Such conditions likely increase the abundance of enzootic mosquito vectors of WNV, the most important of which is *Cx. tarsalis.* When these mosquitoes become sympatric with large numbers of competent avian hosts the ensuing interaction during blood feeding results in WNV amplification.

Overall, spring conditions in western Colorado are wetter than eastern Colorado, due to the high-elevation Rocky Mountains, which generate a large winter snowpack. Water availability may not be a crucial factor limiting vector mosquito activity and abundance in western Colorado. Instead, the colder conditions in the mountains during spring may constrain vector activity and virus amplification. Such possible temperature-limited virus amplification and transmission dynamics require further exploration.

Throughout all of Colorado, dry summer conditions may favor *Cx. pipiens* abundance and infection with WNV. *Culex pipiens* readily exploits eutrophic larval habitats [23], which are favored by dry conditions. While precipitation is the principal source of water to the land surface, irrigation can substantially increase or redistribute land surface wetness, depending on the irrigation practice employed, particularly during times of drought. Throughout Colorado, flood irrigation during dry summers potentially creates a wealth of new vector breeding habitats. The combination of naturally dry summer conditions, further drained riparian zones, and flooded fields may provide both *Cx. tarsalis* and *Cx. pipiens* with additional breeding habitats [9].

In this study, we can only speculate on these issues as we do not have the comprehensive species-specific mosquito population and mosquito and avian infection data needed to investigate such habitat specific effects. Further analyses with such data are needed to determine the relative importance of irrigational effects and natural hydrologic variability at the land surface on vector mosquito abundance and WNV transmission.

Future studies should also examine the effects of other environmental variables on WNV transmission. Temperature variability can alter the extrinsic incubation period of WNV in vector mosquitoes and consequently affect rates of amplification and transmission. A more complete analysis would include temperature and other meteorological variables, as well as hydrology, the human WN case data, and comprehensive entomological, avian, and virological data.

### 4.3. WNV Prediction

At any given locality, WNV amplification and the resulting increased transmission risk may be predicted from certain environmental conditions. Hydrologic conditions, temperature, humidity, and land use practices may influence transmission dynamics. Biological conditions, including vector and avian host population size, reproductive success, local flight, roosting, and dispersal activity, and human behaviors, such as time spent outdoors and use of repellants, must also align. Given all of the potential factors that can modulate WNV activity the strong hydrologic basis for virus transmission, especially in eastern Colorado, is remarkable.

For eastern Colorado, where the linkage between hydrology and human WN cases appears more robust, the best-fit model is based on April and August hydrologic conditions. However, by including August RZSM as an explanatory variable, model usefulness is somewhat limited, as August is the peak month of human WN cases in Colorado. That is, this model would predict high numbers of local human WN cases after most of those cases had already manifest. Models that use July RZSM as an explanatory variable are nearly as accurate and may prove more useful for predicting human risk. Alternatively, observations of spring hydrologic conditions by themselves could be used to provide an early indication of increased human WN transmission risk. In addition, as skillful seasonal forecasts of land surface hydrologic conditions are developed (e.g., [24]), predictions of August RZSM conditions could be coupled with observations of April conditions to forecast human risk. Further research is needed to explore the reliability and predictive skill of such forecasts.

Recent work, using similar data from Colorado, developed a static model of human WN risk based on a number of environmental variables [25]. This model does not evolve in time, but rather predicts the 2002–2006 spatial distribution of WNV transmission in Colorado based on factors such as local long-term average (1961–1990) monthly temperature and precipitation.

The findings presented here use contemporaneous conditions that indicate hydrology could be employed to support the surveillance and control of WNV activity in Colorado *in both space and time,* particularly in eastern Colorado. Ideally, RZSM conditions would be modeled and monitored in real-time and even forecast through a coupling of the Mosaic model with weather and climate forecast models. The hydrologic conditions would then be used to identify ‘risk hotspots’ where vector control and public health interventions would be focused.

## Figures and Tables

**Table 1.** Annual numbers of human WN cases for eastern and western Colorado.

**Location**	**2002**	**2003**	**2004**	**2005**	**2006**	**2007**
**All Colorado**	14	2,947	291	106	345	576
**Eastern Colorado (105.5−102 °W)**	14	2,864	91	92	255	518
**Western Colorado (109−106 °W)**	0	79	199	14	90	58

**Table 2.** Eastern Colorado SAR GLM results with an inverse distance weighting matrix. All parameter estimates are significant at p < 0.0001. Parameter estimates are in units of WN cases per capita per kg/m^2^. The SAR framework reduces model residual spatial autocorrelation to non-significant levels, as assessed by comparing each model residual Moran I statistic to a bootstrapped distribution of Moran I statistics for random permutations of those model residuals. The expected Moran I statistic value for this sample size is −0.0002. The best-fit model is shown in bold.

**Spring Month**	**Summer Month**	**Spring Estimate**	**Spring T Statistic**	**Summer Estimate**	**Summer T Statistic**	**Dev.**	**Moran I Stat.**	**Moran Prob**
March	July	0.044 (0.0022)	19.97	−0.063 (0.0041)	−15.26	3.24e5	−0.0000	0.578
March	August	0.044 (0.0021)	21.34	−0.060 (0.0035)	−16.87	3.18e5	−0.0000	0.578
March	Sept.	0.043 (0.0025)	16.90	−0.029 (0.0042)	−6.92	3.53e5	−0.0000	0.585
April	July	0.049 (0.0021)	23.39	−0.073 (0.0039)	−18.83	3.17e5	−0.0004	0.429
**April**	**August**	**0.051 (0.0020)**	**26.03**	**−0.075 (0.0035)**	**−21.54**	**3.04e5**	−0.0000	**0.570**
April	Sept.	0.043 (0.0028)	15.46	−0.028 (0.0043)	−6.62	3.59e5	−0.0000	0.606
May	July	0.048 (0.0029)	16.54	−0.075 (0.0048)	−15.80	3.38e5	−0.0001	0.577
May	August	0.065 (0.0033)	19.63	−0.094 (0.0050)	−18.78	3.11e5	−0.0000	0.571
June	July	0.039 (0.0040)	9.73	−0.064 (0.0053)	−12.10	3.66e5	−0.0000	0.573
June	August	0.047 (0.0038)	12.40	−0.068 (0.0046)	−14.86	3.54e5	−0.0000	0.591

**Table 3.** Western Colorado SAR GLM results with an inverse distance weighting matrix. Parameter estimates are in units of WN cases per capita per kg/m^2^. The SAR framework reduces model residual spatial autocorrelation to non-significant levels, as assessed by comparing each model residual Moran I statistic to a bootstrapped distribution of Moran I statistics for random permutations of those model residuals. The expected Moran I statistic value for this sample size is −0.0011. The best-fit model is shown in bold.

**Month**	**Estimate**	**T Statistic**	**Significance**	**Deviance**	**Moran I Stat.**	**Moran Prob**
March	−0.0079 (0.0045)	−1.76	0.0788	4.07e4	0.0001	0.633
April	−0.0122 (0.0044)	−2.79	0.0053	4.00e4	0.0001	0.624
May	−0.0191 (0.0067)	−2.87	0.0042	3.98e4	0.0001	0.622
**June**	**−0.0464 (0.0122)**	**−3.80**	**0.0002**	**3.91e4**	**0.0000**	**0.617**
July	−0.0443 (0.0124)	−3.56	0.0004	3.94e4	0.0000	0.626
August	−0.0423 (0.0116)	−3.66	0.0003	3.93e4	0.0000	0.223
